# MonoDFNet: Monocular 3D Object Detection with Depth Fusion and Adaptive Optimization

**DOI:** 10.3390/s25030760

**Published:** 2025-01-27

**Authors:** Yuhan Gao, Peng Wang, Xiaoyan Li, Mengyu Sun, Ruohai Di, Liangliang Li, Wei Hong

**Affiliations:** 1 School of Electronics Information Engineering, Xi’an Technological University, Xi’an 710021, China; gaoyuhan@st.xatu.edu.cn (Y.G.); lixiaoyan@xatu.edu.cn (X.L.); diruohai@xatu.edu.cn (R.D.); 2 Development Planning Service, Xi’an Technological University, Xi’an 710021, China; 3 School of Optoelectronic Engineering, Xi’an Technological University, Xi’an 710021, China; 4 School of Mechanical and Electrical Engineering, Xi’an Technological University, Xi’an 710021, China; liangliangli_xatu@st.xatu.edu.cn (L.L.);hongwei@st.xatu.edu.cn (W.H.)

**Keywords:** deep learning, monocular 3D detection, depth estimation

## Abstract

Monocular 3D object detection refers to detecting 3D objects using a single camera. This approach offers low sensor costs, high resolution, and rich texture information, making it widely adopted. However, monocular sensors face challenges from environmental factors like occlusion and truncation, leading to reduced detection accuracy. Additionally, the lack of depth information poses significant challenges for predicting 3D positions. To address these issues, this paper presents a monocular 3D object detection method based on improvements to MonoCD, designed to enhance detection accuracy and robustness in complex environments. In order to effectively obtain and integrate depth information, this paper designs a multi-branch depth prediction with weight sharing module. Furthermore, an adaptive focus mechanism is proposed to emphasize target regions while minimizing interference from irrelevant areas. The experimental results demonstrate that MonoDFNet achieves significant improvements over existing methods, with AP3D gains of +4.09% (Easy), +2.78% (Moderate), and +1.63% (Hard), confirming its effectiveness in 3D object detection.

## 1. Introduction

**Figure 1:**
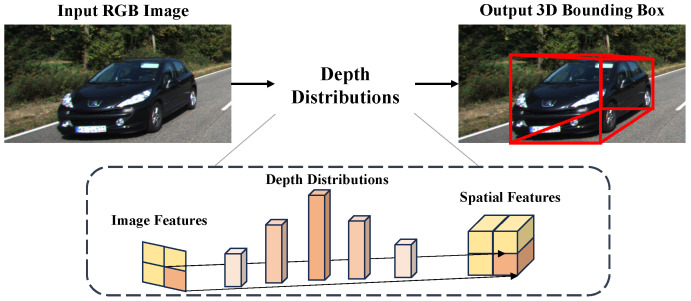
Schematic of monocular 3D object detection.

Monocular 3D object detection is a fundamental task in computer vision with applications in autonomous driving, robotics, and augmented reality. Compared to stereo vision or LiDAR-based methods, monocular 3D detection reduces hardware costs, making it widely used in practical scenarios [1]. Monocular 3D object detection estimates the 3D properties of objects from a single RGB image. The lack of depth information in monocular images poses challenges for accurate 3D detection. To clearly illustrate the core principle of monocular 3D object detection, we provide a schematic diagram in Figure 1.

Monocular 3D detectors have been developed through diverse methodologies, each addressing specific challenges and offering distinct advantages [2]. Table 1 provides a comparative overview of the key categories of monocular 3D detectors, highlighting their representative methods, core characteristics, and associated limitations. This categorization provides insight into the field’s progress and highlights areas for improvement.

As shown in Table 1, each category of monocular 3D detectors has its strengths and weaknesses. Anchor-based [9] methods excel in recall but suffer from computational complexity, while anchor-free [10] and center-based methods offer efficient solutions for real-time applications. Transformer-based detectors introduce powerful global context modeling but at the cost of higher computational demands. Depth-guided and knowledge distillation-based methods effectively bridge gaps between monocular and LiDAR-based detection or enhance model efficiency, but their performance often depends on the quality of depth estimation or teacher models. These insights emphasize the need for frameworks that balance accuracy, efficiency, and robustness in challenging scenarios.

Moreover, accurate depth estimation plays a pivotal role in monocular 3D object detection, as it is essential for predicting the three-dimensional positions of objects [11]. Various depth estimation techniques have been developed to address challenges like depth ambiguity, occlusion, and lighting variations. Table 2 summarizes the key depth estimation approaches, their representative methods, core characteristics, and practical applications, providing a comprehensive overview of their contributions to this field.

As summarized in Table 2, depth estimation techniques have evolved significantly to address the limitations of monocular 3D detection. Self-supervised methods enable scalable deployment by eliminating the need for labeled datasets, while multi-scale approaches enhance depth estimation accuracy across varying object sizes. Diffusion-based techniques and Neural Radiance Fields (NeRFs) provide robust depth estimation [17] in complex scenarios, such as occlusion and extreme lighting conditions. Furthermore, uncertainty estimation methods improve prediction reliability in safety-critical applications like autonomous driving. These advancements highlight depth estimation as a key foundation for more accurate and robust monocular 3D detectors [18]. Further innovations are needed to balance accuracy, efficiency, and real-time performance in varied environments.

Recent advancements have leveraged various depth estimation techniques, including self-supervised methods like Monodepth, multi-scale approaches such as DORN, and diffusion-based methods like MonoDiff. These techniques have significantly improved depth accuracy and robustness in challenging scenarios, though challenges like occlusion and depth ambiguity persist.

MonoCD [19] was proposed in 2024, which focuses on improving feature representation and depth estimation, aiming to bridge the gap between image-based methods and more hardware-intensive solutions. It utilizes advanced feature extraction and depth estimation techniques [20] to generate reliable 3D bounding boxes from a single image. Despite achieving encouraging results, there are still limitations that need to be addressed, such as insufficient accuracy in complex environments and challenges in occlusion handling.

In order to compensate for the shortcomings of the MonoCD network, this article proposes some modifications and improvements to its framework. Specifically, we introduce features such as multi-branch depth prediction [21] to enhance feature representation and orthogonal feature transformation, in order to better obtain spatial relationships [22]. These modifications aim to enhance the robustness and accuracy of monocular 3D detection, particularly in challenging scenarios. By building upon the MonoCD baseline, we aim to demonstrate significant improvements in both quantitative metrics and qualitative performance [23], contributing to the advancement of monocular 3D detection research.

We propose MonoDFNet, a novel monocular 3D detector integrating adaptive target localization, depth-aware focus fusion, and multi-scale depth feature integration to enhance prediction accuracy. The main contributions of this paper are summarized as follows:

(1) We propose a multi-branch depth prediction with weight sharing module, which efficiently integrates depth features across multiple scales. Shared convolutional layers reduce the parameter count while maintaining diverse feature representations. Each branch independently captures scale-specific depth information, enhancing the network’s adaptability to varying object sizes and distances. This multi-branch fusion improves depth estimation accuracy without requiring additional data.
(2) We design a depth awareness and adaptive focus fusion mechanism that integrates a novel adaptive focus mechanism, enhancing the network’s ability to dynamically adjust its attention to important depth features, especially under complex background conditions. This significantly improves the discrimination power of the depth prediction in ambiguous regions.
(3) We make some adjustments to the original network’s strategy that enhance the precision of object localization by employing Gaussian-based heatmap generation combined with a learning rate warm-up schedule. This approach mitigates issues associated with premature convergence and improves the learning capabilities of the network in challenging scenarios.

The remainder of this paper is organized as follows: Section 2 introduces the proposed MonoDFNet framework, detailing its adaptive focus mechanism and multi-branch depth prediction with weight sharing. Section 3 describes the experimental setup, including datasets, evaluation metrics, and experimental results, followed by an ablation study and visualization analysis. Finally, Section 4 concludes this paper with a summary of findings and potential future directions.

## 2. Approach

### 2.1. MonoDFNet

MonoDFNet is an improved network built on MonoCD, specifically for monocular 3D object detection. It includes several optimizations to enhance detection accuracy, particularly in depth estimation. The main innovation of MonoDFNet lies in multi-scale deep feature fusion and the adaptive attention mechanism. In addition, some parameter settings of MonoCD have been adjusted, with detailed parameter configurations provided in Section 3.3. MonoDFNet modifies both the learning strategy [24] and the heatmap generation method. Adjustments to the focus loss parameters (α, β, γ) enhance the network’s handling of class imbalance, improving detection in complex scenes with varying foreground–background distributions.

The Focal Loss function is widely used to address class imbalance issues in object detection tasks. Two common variations are Vanilla Focal Loss (V-F Loss) and Penalty-Reduced Focal Loss (P-R-F Loss). V-F Loss enhances the model’s focus on hard-to-classify examples by reducing the contribution of well-classified samples. α balances the importance of positive and negative samples. γ adjusts the degree of focus on hard samples. Common values range from 1.0 to 3.0, with 2.0 being the most frequently used. Typical values range from 0.25 to 0.75. The formula for V-F Loss is:

(1) $$FL(p_{t})=-\alpha_{t}{\left(1-p_{t}\right)}^{\gamma }\log(p_{t})$$

where $p_{t}$ represents the predicted probability for the true class, which is the model’s confidence score for a sample being correctly classified. Specifically, $p_{t}$ is obtained from the output of the network’s final layer, typically after applying a sigmoid or softmax activation function, and it ranges between 0 and 1. A higher value of $p_{t}$ indicates greater confidence in the correct classification of the sample, while a lower value implies that the model is uncertain or incorrect in its prediction. $\alpha_{t}$ is the class balancing parameter, used to address imbalanced data by giving higher weights to underrepresented classes. γ is the focusing parameter that adjusts the weight assigned to hard-to-classify examples by emphasizing samples where $p_{t}$ is small (i.e., low confidence).

In addition to the standard Focal Loss, MonoDFNet also utilizes Penalty-Reduced Focal Loss, which introduces an additional β parameter to further regulate the network’s focus on difficult-to-classify examples. α typically ranges from 0.5 to 2.5, controlling the balance between classes. β adjusts the influence of the penalty term, with values usually ranging from 3.0 to 5.0. The Penalty-Reduced Focal Loss function can be expressed as:

(2) $$PRFL(p_{t})=-\alpha_{t}{\left(1-p_{t}\right)}^{\gamma }\log(p_{t})+\beta_{t}{\left(1-p_{t}\right)}^{2}$$

The β parameter introduces an additional penalty term that intensifies the punishment on misclassified samples, enabling quicker correction during training. This adjustment enhances the network’s overall performance, particularly in scenarios with a pronounced imbalance between easily classified and challenging samples.

**Figure 2:**
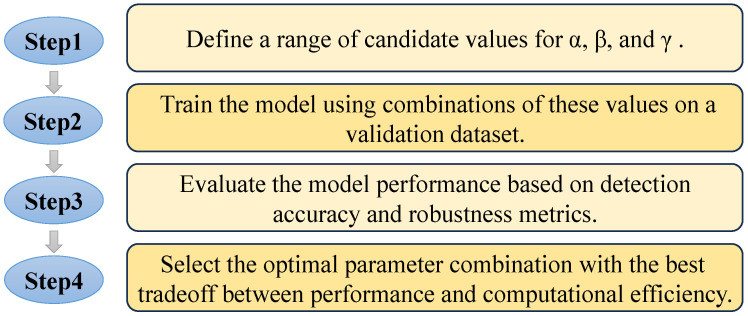
Workflow for parameter selection of *α*, *β*, and *γ*.

To determine the optimal values for the parameters *α*, *β*, and *γ* in the Focal Loss function, we performed a series of experiments with different parameter combinations on the training dataset in Section 3.4. Each parameter was tuned based on its influence on class imbalance handling, with *α* balancing the contribution of positive and negative samples, *β* penalizing misclassified samples, and *γ* focusing on hard-to-classify examples. The final selected values were those that maximized detection accuracy while maintaining robustness across different scenarios. The determination method of these three parameters is shown in Figure 2.

**Figure 3:**
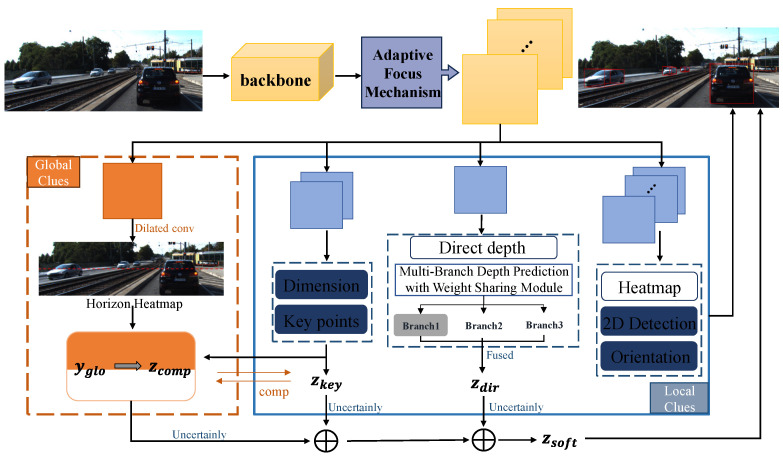
MonoDFNet architecture overview.

The overall framework of the network is shown in Figure 3.

The overall process of the network is as follows. Firstly, the monocular image is input and subjected to backbone feature extraction. Then, an adaptive attention mechanism is introduced to process the features extracted from the backbone and dynamically adjust their spatial distribution. This mechanism selectively emphasizes regions with key depth information while suppressing irrelevant areas, enhancing adaptability in complex scenarios.

Subsequently, the extracted features are assigned to multiple prediction heads, namely, Global Clues and Local Clues. The Global Clues module captures overall scene information, such as depth distribution and horizontal line features, while the Local Clues module refines local target predictions, providing precise boundaries, sizes, and poses.

In the Local Clues module, the network further integrates multi-scale deep features through the multi-branch depth prediction module. Through the weight-sharing mechanism, this module can effectively capture depth information at different scales while reducing the number of parameters to maintain computational efficiency. This enables the network to more accurately detect target objects of different distances and sizes and to maintain robustness in complex scenes.

Finally, after integrating Global Clues and Local Clues, the network generates the final 3D object detection results, including the 3D position, size, and orientation of the target.

### 2.2. Adaptive Focus Mechanism

**Figure 4:**
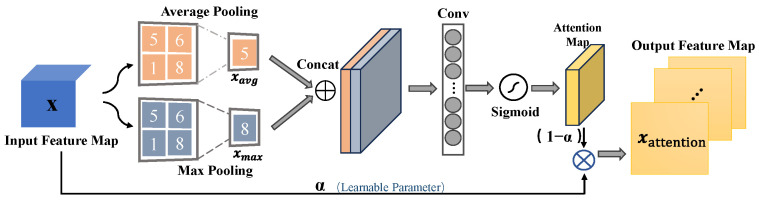
Architecture of the adaptive focus mechanism.

MonoDFNet introduces an adaptive focus mechanism to enhance feature extraction by emphasizing important spatial regions and suppressing less relevant ones, as shown in Figure 4. The mechanism uses a spatial attention module to calculate an attention map from the input feature map [25], allowing dynamic focus adjustment based on spatial feature distribution.

Average pooling and max pooling operations generate the attention map, capturing critical information across channels. Mathematically, the spatial attention map $A_{spatial}$ is derived as follows:

Let $x_{avg}$ and $x_{\max}$ represent the average and max pooled feature maps over the channels of the input feature map *x*, respectively:

(3) $$x_{avg}=AvgPool(x,dim=1)$$

(4) $$x_{\max}=MaxPool(x,dim=1)$$

These pooled feature maps are concatenated and passed through a convolutional layer to compute the spatial attention map:

(5) $$A_{spatial}=\sigma (Conv2D(\left[x_{avg},x_{\max}\right],1,kernel\_size=9,padding=4))$$

where σ represents the sigmoid activation function. The activation normalizes attention map values between 0 and 1, helping the network focus on key spatial regions.

The attention map is applied to the original feature map *x*, where a learnable parameter *α* balances the original and attention-weighted features. The final output feature map $x_{attention}$ is computed as:

(6) $$x_{attention}=\alpha \cdot x+(1-\alpha )\cdot A_{spatial}$$

This equation represents how the input feature map *x* is combined with the spatial attention map to form the final attention-weighted feature map. By learning the parameter *α*, the network can flexibly adjust how much attention is applied to the spatial features, allowing it to better focus on the most relevant areas during the object detection and depth estimation tasks.

**Figure 5:**
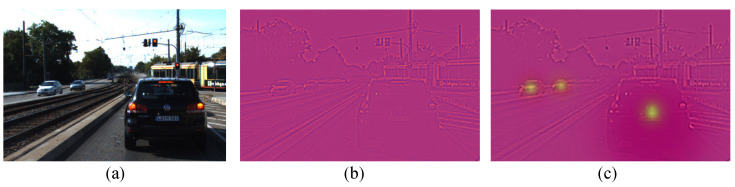
Comparison of feature map processing effects.

Figure 5 illustrates a comparison of the feature map before and after processing. Specifically, Figure 5a represents the original input image, Figure 5b shows the feature map generated by the backbone network of the original model, and Figure 5c depicts the feature map enhanced through the adaptive focus mechanism.

The adaptive focus mechanism enhances important spatial features while reducing noise from irrelevant regions, improving accuracy and stability.

### 2.3. Multi-Branch Depth Prediction with Weight Sharing Module

**Figure 6:**
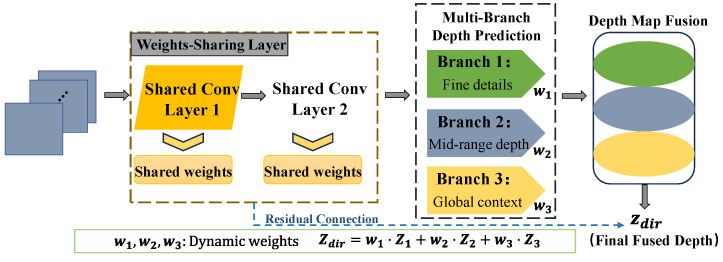
Architecture of the multi-branch depth prediction with weight sharing module.

The multi-branch depth prediction with weight sharing module in MonoDFNet integrates multi-scale depth information and reduces parameters through weight sharing, as illustrated in Figure 6. This design reduces computational cost and improves depth prediction accuracy by capturing variations across different depth scales [26]. The design of this module is grounded in the principle that objects at different distances exhibit distinct spatial characteristics and contextual dependencies. Near objects require fine-grained details for accurate depth estimation, whereas far objects rely more on global context and scene-level information. By integrating multi-scale depth information, this module ensures that each branch specializes in processing depth features at a specific scale, effectively addressing this inherent complexity. Furthermore, the weight-sharing mechanism reduces redundant computations by sharing parameters across branches, which not only minimizes the model’s complexity but also enforces consistency in the extracted features. This consistency is crucial for enabling the dynamic fusion strategy to effectively combine multi-scale features, as it ensures compatibility across branches while maintaining computational efficiency.

**Figure 7:**
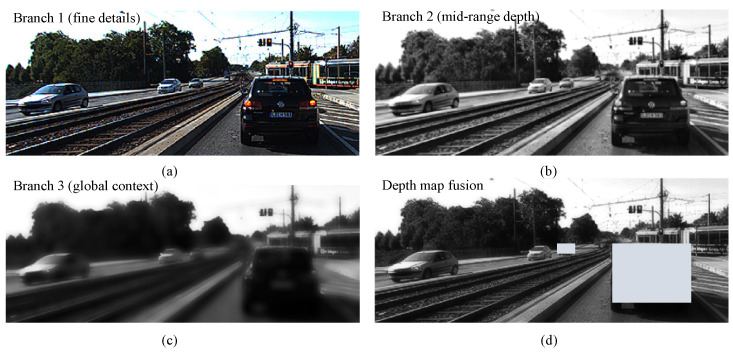
Feature visualization and depth map fusion in the multi-branch architecture.

This module begins with shared convolutional layers that extract global features from input feature maps. The shared convolutional layers serve as a foundational feature extractor by providing a unified representation of the input data. This ensures that each subsequent branch starts from a consistent global feature map, which is critical for maintaining the integrity of the depth prediction process. Branch 1 focuses on extracting high-frequency details that are essential for near-object depth estimation, such as edges and textures. In contrast, Branch 2 is designed to process intermediate-depth information, providing a smooth transition between fine details and the global context. Branch 3 captures low-frequency, large-scale features that are indispensable for distant-object estimation. This hierarchical structure reflects the fundamental divide-and-conquer approach, where the depth prediction task is decomposed into specialized sub-tasks for more efficient and accurate processing. The ability to independently process and combine these features is further enhanced by the dynamic fusion mechanism, which adapts the contributions of each branch based on the scene’s depth distribution. This adaptability ensures that the module can generalize effectively across diverse and complex environments. Figure 7 visualizes the contributions of each branch, highlighting improvements in depth prediction across different scales.

The figure shows the functionality of each branch. Branch 1 captures fine details like vehicle edges and road features. Branch 2 enhances mid-range contrast for smoother transitions. Branch 3 preserves the global context through controlled blurring. The fused depth map dynamically combines branch outputs, highlighting key areas such as vehicles for enhanced depth perception. Objects at varying distances exhibit distinct spatial resolutions and context dependencies, which single-branch configurations struggle to capture. The multi-branch design ensures that near objects benefit from high-frequency details, while distant objects are supported by global contextual understanding. By assigning adaptive weights to each branch based on scene-specific requirements, the fused depth map achieves a balanced representation that highlights both fine details and global structures. This design ensures that the module can flexibly adapt to varying scene complexities, providing robust and accurate depth predictions even in challenging scenarios.

The outputs from the three branches are fused using a dynamic strategy that adaptively balances their contributions based on scene requirements. The shared feature map $x_{shared}$ is computed as:

(7) $$x_{shared}=f_{shared}(x)=\sigma (W_{shared2}\cdot \sigma (W_{shared1}\cdot x+b_{shared1})+b_{shared2})$$

where $W_{shared1}$ and $W_{shared2}$ are the weight matrices, $b_{shared1}$ and $b_{shared2}$ are biases, and σ represents the activation function. Each branch processes $x_{shared}$ independently:

(8) $$x_{branch_{i}}=f_{branch_{i}}(x_{shared})=\sigma (W_{branch_{i}}\cdot x_{shared}+b_{branch_{i}})\;\mathrm{for}\;i=1,2,3$$

where $W_{branch_{i}}$ and $b_{branch_{i}}$ represent the weights and biases for each branch.

The dynamic fusion strategy combines the branch outputs, where each branch’s contribution is weighted by a learnable parameter $a_{i}$, normalized using SoftMax:

(9) $$w_{i}=\frac{\exp(\alpha_{i})}{\sum_{j=1}^{3}\exp(\alpha_{j})},i=1,2,3$$

The final depth map $D_{fused}$ is obtained by weighting the branch outputs:

(10) $$D_{fused}=\sum_{i=1}^{3}w_{i}\cdot D_{branch_{i}}$$

This strategy ensures that the model adaptively prioritizes fine details or the global context based on the scene’s complexity.

Residual connections are added to both the shared and branch layers to improve gradient flow and stabilize training. For the shared layers, the residual connection is defined as:

(11) $$x_{residual1}=W_{residual1}\cdot x+b_{residual1}$$

and is added to the shared feature map:

(12) $$x_{shared\_final}=x_{shared}+x_{residual1}$$

These connections preserve low-level features and mitigate gradient vanishing, particularly in deeper layers. The module achieves computational efficiency by reducing parameters through weight sharing and optimizing memory usage via channel reduction in each branch. By combining depth features from multiple scales, the module provides accurate and robust depth prediction across diverse scenarios.

## 3. Experiments

### 3.1. Dataset

The experiments used the widely recognized KITTI 3D Object dataset. The dataset comprises 7481 images for training and 7518 images for testing. Since the ground truth annotations for the test set are not publicly available, we followed standard practice and split the 7481 training images into two sets: 3712 images for training and 3769 images for validation [27]. Each object category was divided into three difficulty levels—Easy, Moderate, and Hard—based on object height, truncation, and occlusion in the 2D image.

### 3.2. Evaluation Metrics

In line with previous work, we used Average Precision (AP) as the primary metric to evaluate the performance of our model. AP3D measures precision in 3D space, while APBEV evaluates precision from a bird’s eye view (BEV). These metrics [28] provide a comprehensive assessment of both the spatial accuracy of detected objects and their alignment with ground truth in a top-down perspective.

Average accuracy was calculated at 40 recall positions following standard evaluation protocols. AP is defined as the area under the precision–recall curve, which can be expressed as:

(13) $$AP=\int_{0}^{1}P(r)dr$$

where P(r) represents the precision as a function of recall *r*. This calculation provides a single value that summarizes the model’s ability to balance accuracy and recall across different thresholds.

For the Car category, an Intersection over Union (IoU) threshold was used to determine the true positives of 3D space and BEV. The IOU calculation is as follows:

(14) $$IOU=\frac{Area\;of\;Overlap}{Area\;of\;Union}$$

This threshold requires a significant overlap between the detected object and ground truth to qualify as a correct detection. By using these indicators, we comprehensively evaluated the performance of the model in terms of detection accuracy and localization.

### 3.3. Settings

The hardware configurations and software versions used for the experiments in this paper are listed in Table 3.

Regarding the network parameter setting, in MonoDFNet, the center distribution of the 3D heatmap as replaced with a Gaussian distribution to improve the accuracy and robustness of target localization. A learning rate preheating strategy gradually increased the learning rate early in training, preventing gradient instability and ensuring smoother convergence. Additionally, the focus loss parameters were configured as follows: for V-F-Loss, the optimal settings were α = 0.5 and γ = 2.5; for P-R-F-Loss, the parameters were set to α = 1.5 and β = 3.0. The reason behind these parameter choices will be analyzed in Section 3.6. All other parameter settings follow those of MonoCD.

### 3.4. Experimental Results and Analysis

We compare MonoDFNet with state-of-the-art monocular 3D detection methods, as shown in Table 4, to demonstrate its effectiveness. The best results are highlighted in bold, and the second-best results are underlined. The table lists each model along with the additional data used, if any, and compares their performance on the AP3D and APBEV metrics across different difficulty levels (Easy, Moderate, and Hard) on the KITTI dataset.

The data in the table clearly shows that MonoDFNet, our model, does not rely on additional data sources (such as LiDAR or depth maps) and surpasses all other methods, including the baseline model MonoCD, across all difficulty levels. Specifically, for AP3D, our model achieves accuracies of 25.71%, 19.07%, and 15.96% for the Easy, Moderate, and Hard levels, respectively; for APBEV, it achieves accuracies of 33.56%, 24.52%, and 21.01%, respectively. These results indicate improvements of +4.09% (Easy), +2.78% (Moderate), and +1.63% (Hard) over the second-best model in AP3D and improvements of +3.98%, +1.95%, and +1.39% in APBEV.

The results show that the proposed enhancements improve depth estimation and feature extraction under challenging conditions, without relying on additional sensor data. MonoDFNet achieves competitive inference speed (as shown in the Time column), ensuring that the improvements do not increase latency.

To validate the method’s effectiveness and generalizability, we integrated the proposed modules into two widely used baselines (MonoDLE and MonoRCNN). Table 5 presents the comparative experimental results on the KITTI dataset using the AP3D and APBEV metrics across different difficulty levels (Easy, Moderate, and Hard). The improvement achieved by incorporating our method is highlighted in the “Improvement” rows.

As shown in Table 5, incorporating the proposed method consistently improves the performance of both MonoDLE and MonoRCNN across all difficulty levels for both the AP3D and APBEV metrics. The enhancements are particularly significant under the “Easy” setting, with improvements of up to +2.82% for AP3D and +2.51% for APBEV. These results demonstrate that the proposed method effectively enhances the depth estimation and feature extraction capabilities of existing monocular 3D detectors, validating its effectiveness and versatility in monocular 3D object detection tasks.

### 3.5. Visualization Results and Analysis

**Figure 8:**
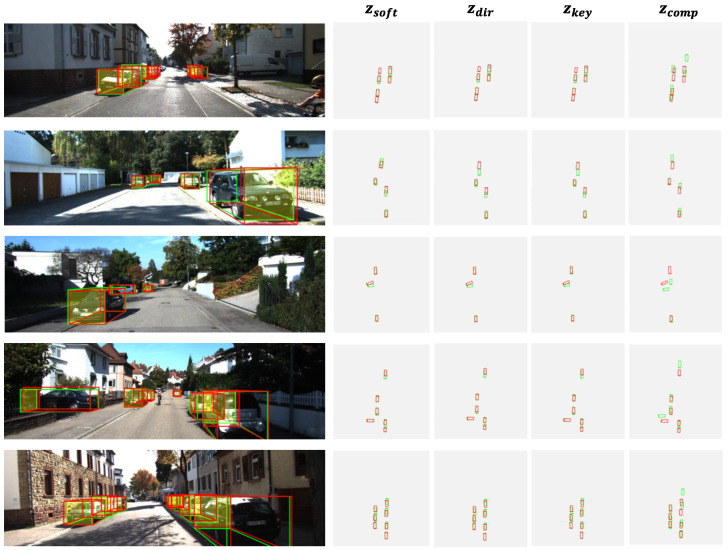
Detection visualization results. $z_{soft}$ : simple fusion based on SoftMax; $z_{dir}$ dynamic fusion strategy; $z_{key}$: depth fusion based on key features; $z_{comp}$: final fusion results combining multiple methods.

In this section, we present and analyze the visualization results of the proposed method, showing how the model performs on various depth estimation strategies, as shown in Figure 8. In each row, we provide a final front-view visualization (left) and four bird’s-eye-view (BEV) visualizations (right). The visualizations are divided into four distinct depth estimation types, i.e., $z_{soft}$, $z_{dir}$, $z_{key}$, and $z_{comp}$, which correspond to different approaches within the depth prediction process. Each column represents the detection results of the bounding boxes on a specific depth estimation type, and each row displays a different scene for comparison. Red bounding boxes represent ground truth, while green bounding boxes represent the model’s predictions.

The alignment between the red and green boxes illustrates the effectiveness of each method in capturing depth information. The visualization results demonstrate that our model achieves accurate predictions in long-range, short-range, dense, and sparse environments. This highlights its exceptional robustness in complex urban scenarios, even under conditions of occlusion, varying object densities, and intricate road geometries. From the first and fifth rows, it is evident that the model performs well in sparse close-range scenes and occluded long-range cases. In the second row, the model accurately detects vehicles under complex lighting conditions with alternating strong light and shadows. In the third row, it successfully identifies distant vehicles and fine details in occluded environments. The fourth row further shows that the model can effectively detect multiple adjacent vehicles in dense and complex urban environments while maintaining excellent prediction performance. These results demonstrate the model’s reliability and practicality across different application scenarios.

The visualization results confirm the advantages of the proposed depth prediction strategy, particularly with $z_{soft}$ providing superior depth estimation and localization. Progressing through different depth methods highlights the benefits of the multi-branch approach, where each method addresses specific object characteristics. This depth diversity improves detection accuracy and robustness across diverse scenes, validating the effectiveness of the adaptive focus and multi-scale fusion mechanisms integrated into the model.

### 3.6. Ablation Study

An ablation study, shown in Table 6, was conducted to validate the effectiveness of each module and improvement. The performance was measured using Average Precision for 3D Object Detection (AP3D) and bird’s eye view (APBEV) under three difficulty levels: Easy, Moderate (Mod.), and Hard.

With each enhancement, the detection accuracy improves incrementally. Model A achieves AP3D scores of 21.62%, 16.29%, and 14.33% for the Easy, Moderate, and Hard cases. Adding module B improves AP3D by +1.57%, +1.60%, and +0.63%, respectively. Incorporating module C enhances depth estimation, adding +0.87%, +0.89%, and +0.21% in AP3D for the three difficulty levels. Finally, with module D, the model achieves the highest accuracy, adding +1.65%, +0.29%, and +0.79% to reach AP_3D_ scores of 25.71%, 19.07%, and 15.96% for the Easy, Moderate, and Hard cases.

For AP_BEV_, model A begins with scores of 29.41%, 22.57%, and 19.62% for the three levels. Module B contributes an increase of +0.68%, +0.75%, and +0.42%. Adding module C further boosts AP_BEV_ by +1.93%, +0.53%, and +0.69%. Adding module D improves APBEV by +1.54%, +0.67%, and +0.28%, achieving scores of 33.56%, 24.52%, and 21.01% for the Easy, Moderate, and Hard cases.

The results demonstrate that each component positively contributes to performance, yielding measurable gains in both AP3D and APBEV.

Table 7 presents the results of the ablation study conducted to evaluate the impact of different parameter combinations (α, β, γ) in the Vanilla Focal Loss (V-F-Loss) and Penalty-Reduced Focal Loss (P-R-F-Loss) functions. The data marked as “baseline” in Table 7 correspond to the parameter values originally set by the MonoCD model. These include (α = 0.25, γ = 2.0) for Vanilla Focal Loss (V-F Loss) and (α = 2.0, β = 4.0) for Penalty-Reduced Focal Loss (P-R-F Loss). Based on these baseline configurations, we performed a series of small-range adjustments to explore the parameter space. Specifically, for V-F Loss, α was incrementally increased by 0.25, and γ by 0.5, while retaining the original baseline values as the lower bounds. For P-R-F Loss, both incremental and decremental adjustments were made, with α changed by ±0.5 and β by ±1.0. The optimal parameter values for V-F Loss and P-R-F Loss are highlighted in bold.

The ablation results in Table 7 highlight the sensitivity of both loss functions to parameter tuning. For V-F-Loss, the optimal configuration is achieved with α = 0.5 and γ = 2.5, resulting in the highest AP3D and APBEV scores across all difficulty levels. Similarly, for P-R-F-Loss, the configuration with α = 1.5 and β = 3.0 outperforms other settings, demonstrating better balance in handling class imbalance and difficult examples. The optimal values are highlighted in bold in the table for clarity. These findings emphasize the importance of careful parameter tuning to enhance model robustness and detection accuracy.

In the experiment, due to the small initial value of α in the original model, it was observed that slightly increasing its value led to an improvement in accuracy. However, further increasing α led to a decrease in accuracy, which helped determine the optimal parameter settings for this network. Similarly, for P-R-F Loss, the two related parameters first simultaneously decreased and then increased. It was observed that slightly reducing their values improved model accuracy.

We further conducted an internal ablation study on the multi-branch depth prediction with weight sharing module (C). The results are shown in Table 8. The ablation study on Module C highlights the distinct contributions of its core components—the weight-sharing layer, multi-branch design, and residual connections—to the overall performance improvement. Each component addresses a specific challenge in depth estimation, collectively enhancing the model’s robustness and adaptability across diverse and complex scenarios.

The weight-sharing layer optimizes parameter efficiency by enabling shared feature extraction across all branches. This design reduces model complexity while maintaining consistent and reliable global feature extraction, which forms the foundation for subsequent depth predictions.

The multi-branch design is pivotal in capturing depth variations at different scales. Each branch specializes in processing depth information for near, medium, or distant objects, ensuring the model can adapt to varying scene complexities. Unlike single-branch architectures that struggle to balance fine details and the global context, the multi-branch approach divides the task among specialized sub-networks, effectively handling uneven depth distributions and challenging scenarios, such as distant or partially occluded objects. Furthermore, the dynamic fusion strategy assigns optimal weights to each branch based on the scene, ensuring that predictions are both accurate and contextually relevant.

Residual connections address the critical challenge of gradient flow in deep networks. By directly propagating low-level features across layers, these connections preserve essential information for depth estimation while mitigating gradient vanishing during training. This design ensures the stability of the training process and enhances the network’s ability to refine predictions in complex scenes.

Altogether, each component contributes independently to performance improvements, and the complete module delivers an overall accuracy increase of 1% to 2% in both AP3D and APBEV, validating the effectiveness of the proposed module.

Additionally, to demonstrate the necessity of selecting three branches for depth prediction, Table 9 presents an ablation study on the different numbers of branches. The optimal values are highlighted in bold.

Table 9 highlights the effectiveness of the multi-branch depth prediction design. Compared to the single-branch configuration, the three-branch setup demonstrates significant improvements, particularly under the Hard level, where AP3D increases from 14.33% to 15.49% and APBEV improves from 19.62% to 20.51%. The three-branch configuration is a cornerstone of Module C’s effectiveness. Unlike single- or two-branch setups, which struggle to fully capture multi-scale depth features, the three-branch design comprehensively addresses depth variations across near, medium, and far ranges. This approach is grounded in a fundamental principle of depth estimation: objects at different distances exhibit distinct spatial and contextual characteristics, necessitating a tailored processing strategy. By leveraging complementary information from all three branches, the model achieves a well-balanced representation of fine details and global context. These results underscore the multi-branch approach’s ability to effectively capture depth features across different scales, enabling more accurate predictions for distant objects and complex scenes. The intermediate performance of the two-branch configuration further reinforces the necessity of the complete three-branch design.

## 4. Conclusions

MonoDFNet is an enhanced monocular 3D detection network based on MonoCD, designed to overcome the original model’s limitations in complex environments and occlusion scenarios. The multi-branch depth prediction with weight sharing module uses multiple branches to predict depth at various scales, capturing a comprehensive depth representation. The weight-sharing design minimizes parameter count, improving computational efficiency. The adaptive focus mechanism improves the network’s focus on critical information while reducing the influence of irrelevant details, enhancing detection accuracy. Adjusting the parameters further optimizes the model’s performance. Our extensive experiments thoroughly validate the proposed method. On the KITTI dataset, MonoDFNet achieves notable AP3D improvements of +4.09% (Easy), +2.78% (Moderate), and +1.63% (Hard), along with APBEV gains of +3.98%, +1.95%, and +1.39%, without requiring additional data sources like LiDAR or depth maps. The visualization results demonstrate MonoDFNet’s robustness in managing occlusions, varying object densities, and challenging lighting conditions.

## Figures and Tables

**Table 1:** Categories of monocular 3D detectors and their characteristics.

Category	Representative Methods	Characteristics	Limitations
Anchor-based	M3D-RPN [3]	High recall, predefined anchor boxes for region proposals	High computational cost due to multiple anchor configurations
Anchor-free	MonoFlex [4]	Flexible regression strategies, efficient and fast	Potentially less robust in highly cluttered scenes
Center-based	CenterNet [5]	Predicts object centers and related attributes, balances speed and accuracy	May struggle with small or occluded objects
Transformer-based	MonoDETR [6]	Leverages attention mechanisms for global context modeling, improves robustness in complex scenes	High computational complexity
Depth-guided	Pseudo-LiDAR [7]	Converts monocular images into point clouds, bridges monocular and LiDAR-based detection	Relies heavily on intermediate depth estimation quality
Knowledge distillation	MonoDistill [8]	Transfers knowledge from LiDAR-based teacher models to lightweight monocular student models	Performance depends on teacher mode quality

**Table 2:** Depth estimation techniques in monocular 3D detection.

Technique	Representative Methods	Characteristics	Applications
Self-supervised	Monodepth [12]	Uses image reconstruction loss, eliminates need for ground truth depth labels	Scalable deployment for real-world scenarios
Multi-scale depth estimation	DORN [13]	Captures global and local depth features to improve accuracy	Useful for objects of varying sizes
Diffusion-based	MonoDiff [14]	Iterative refinement, robust to challenging conditions such as occlusion and varying lighting	Depth estimation under extreme conditions
Uncertainty estimation	MonoDLE [15]	Models confidence in depth predictions, enhances reliability	Safety-critical applications such as autonomous driving
Neural Radiance Fields	MonoNeRF [16]	Enriches depth information through volumetric scene representation	Improves localization in complex 3D scenes

**Table 3:** Experimental configuration.

Items	Detail
Operating System	Ubuntu20.04
GPU	Quadro M6000/PCIe/SSE2 Manufacturer: NVIDIA, Santa Clara, CA, United States
CPU	13th Gen Intel Core i7-13700K × 24 Manufacturer: Intel, Santa Clara, CA, United States
CUDA	10.1
CUDNN	7.6
Python	3.7.0
Pytorch	1.4.0

**Table 4:** Comparative experimental results.

Methods	Venues	Extra Data	Test, AP3D (%)			Test, APBEV (%)			Time (ms)
			Easy	Mod.	Hard	Easy	Mod.	Hard	
DDMP-3D [29]	CVPR2021	Depth	15.63	12.38	9.52	24.06	17.66	13.39	190
Kinematic3D	ECCV2020	Video	14.94	12.33	8.91	22.68	17.29	13.02	130
AutoShape [30]	ICCV2021	CAD	18.56	14.27	11.42	26.25	19.98	15.24	60
DCD [31]	ECCV2022		19.92	16.01	12.37	28.14	21.03	18.35	-
MonoRUn [32]	CVPR2021	LiDAR	15.57	11.92	10.24	24.39	17.05	14.93	80
CaDDN [33]	CVPR2021		15.02	13.05	11.55	24.36	18.72	17.35	640
MonoDTR [34]	CVPR2022		18.21	15.52	12.63	25.43	20.21	17.31	46
SMOKE [35]	CVPRW2020	None	10.04	9.32	7.45	17.21	13.85	12.58	40
MonoDLE	CVPR2021		13.49	11.89	10.03	21.34	18.77	16.02	50
MonoRCNN [36]	ICCV2021		14.52	12.46	10.13	22.16	18.05	14.16	80
MonoFlex	CVPR2021		15.86	13.62	12.12	24.33	19.64	16.90	45
MonoGround [37]	CVPR2022		17.61	14.02	12.78	26.10	20.36	17.69	40
MonoJSG [38]	CVPR2022		20.18	15.84	13.39	28.31	21.01	18.20	53
MonoCon [39]	AAAI2022		18.09	16.16	13.64	17.03	21.99	19.06	36
MonoDETR	ICCV2023		21.14	15.17	13.53	29.58	22.10	18.68	55
MonoCD	CVPR2024		21.62	16.29	14.33	29.41	22.57	19.62	42
MonoDFNet	(Ours)	None	**25.71**	**19.07**	**15.96**	**33.56**	**24.52**	**21.01**	49
Improvement		vs. second-best	+4.09	+2.78	+1.63	+3.98	+1.95	+1.39	-

**Table 5:** Performance comparison of different models enhanced by our method.

Method	Test, AP3D (%)			Test, APBEV (%)		
	Easy	Mod.	Hard	Easy	Mod.	Hard
MonoDLE	13.49	11.89	10.03	21.34	18.77	16.02
+Ours	15.63	12.95	11.45	23.48	19.91	17.13
Improvement	+2.14	+1.06	+1.42	+2.14	+1.14	+1.11
MonoRCNN	14.52	12.46	10.13	22.16	18.05	14.16
+Ours	17.34	14.56	11.82	24.67	19.51	15.98
Improvement	+2.82	+2.10	+1.69	+2.51	+1.46	+1.82

**Table 6:** Ablation experimental results.

	Test, AP3D (%)			Test, APBEV (%)		
	Easy	Mod.	Hard	Easy	Mod.	Hard
Baseline (A)	21.62	16.29	14.33	29.41	22.57	19.62
+Parameter Adjustment (B)	23.19	17.89	14.96	30.09	23.32	20.04
+Multi-Branch Depth Prediction with Weight Sharing Module (C)	24.06	18.78	15.17	32.09	23.85	20.73
+Adaptive Focus Mechanism (D)	25.71	19.07	15.96	33.56	24.52	21.01

**Table 7:** Ablation study on Focal Loss and Penalty-Reduced Focal Loss parameters.

Loss Function	α	β	γ	Test, AP3D (%)			Test, APBEV (%)		
				Easy	Mod.	Hard	Easy	Mod.	Hard
V-F Loss (baseline)	0.25	**-**	2.0	21.62	16.29	14.33	29.41	22.57	19.62
V-F Loss	0.5	-	2.5	**23.86**	**17.59**	**15.58**	**31.10**	**23.37**	**20.22**
V-F Loss	0.75	-	3.0	21.53	16.81	15.17	28.96	22.63	19.37
P-R-F Loss (baseline)	2.0	4.0	-	21.62	16.29	14.33	29.41	22.57	19.62
P-R-F Loss	1.5	3.0	-	**23.31**	**17.15**	**15.82**	**31.22**	**23.64**	**20.45**
P-R-F Loss	1.0	2.0	-	22.21	16.94	14.63	30.38	22.53	19.64
P-R-F Loss	2.5	5.0	-	20.41	16.88	14.16	30.74	22.41	18.92

**Table 8:** Module C ablation experimental results.

	Test, AP3D (%)			Test, APBEV (%)		
	Easy	Mod.	Hard	Easy	Mod.	Hard
Baseline	21.62	16.29	14.33	29.41	22.57	19.62
+Weights Sharing Layer	22.44	16.60	14.59	30.31	22.80	19.67
+Multi-Branch Depth Prediction	23.81	17.33	14.93	31.10	23.37	20.37
+Residual Connection	23.67	18.31	15.49	31.39	23.87	20.51

**Table 9:** Ablation study on the number of branches in Module C.

Method	Test, AP3D (%)			Test, APBEV (%)		
	Easy	Mod.	Hard	Easy	Mod.	Hard
Baseline + Single Branch	21.62	16.29	14.33	29.41	22.57	19.62
Baseline + Two Branches	21.54	16.85	14.72	30.12	23.12	19.97
Baseline + Three Branches	**23.67**	**18.31**	**15.49**	**31.39**	**23.87**	**20.51**

## Data Availability

The data supporting the findings of this study and the source code for the implementation are available at the GitHub repository: https://github.com/Gaoooyhan/MonoDFNet (accessed on 20 November 2024). Additional data that support the findings of this study are available from the corresponding author upon reasonable request.
